# Depressive Pseudodementia with Reversible AD-like Brain Hypometabolism: A Case Report and a Review of the Literature

**DOI:** 10.3390/jpm12101665

**Published:** 2022-10-06

**Authors:** Federico Emanuele Pozzi, Daniele Licciardo, Monica Musarra, Lorenzo Jonghi-Lavarini, Cinzia Crivellaro, Gianpaolo Basso, Ildebrando Appollonio, Carlo Ferrarese

**Affiliations:** 1 Neurology Department, San Gerardo Hospital, 20900 Monza, Italy; 2 Neuropsychology Department, San Gerardo Hospital, 20900 Monza, Italy; 3 Nuclear Medicine Department, San Gerardo Hospital, 20900 Monza, Italy; 4 Neuroradiology Department, San Gerardo Hospital, 20900 Monza, Italy; 5 Milan Center for Neuroscience (NeuroMI), University of Milano-Bicocca, 20126 Milan, Italy

**Keywords:** case report, depression, pseudodementia, FDG-PET, Alzheimer’s disease, reversible hypometabolism

## Abstract

Recent European guidelines recommend using brain FDG-PET to differentiate between Alzheimer’s disease (AD) and depressive pseudodementia (DP), with specific hypometabolism patterns across the former group, and typically normal or frontal hypometabolism in the latter. We report the case of a 74 years-old man with DP (MMSE 16/30), whose FDG-PET visual rating and semiquantitative analysis closely mimicked the typical AD pattern, showing severe hypometabolism in bilateral precuneus, parietal and temporal lobes, and sparing frontal areas, suggesting the diagnosis of moderate AD. Shortly after starting antidepressant polytherapy, he underwent formal NPS testing, which revealed moderate impairment of episodic memory and mild impairment on executive and visuospatial tests, judged consistent with neurodegenerative dementia and concomitant depression. Over the following two years, he improved dramatically: repeated NPS assessment did not show significant deficits, and FDG-PET showed restoration of cerebral metabolism. The confirmation of PET findings via semiquantitative analysis, and their reversion to normality with antidepressant treatment, proved the non-neurodegenerative origin of the initial AD-like FDG-PET abnormalities. We review similar cases and provide a comprehensive analysis of their implications, concluding that reversible FDG-PET widespread hypometabolism might represent a biomarker of pseudodementia. Therefore, we suggest caution when interpreting FDG-PET scans of depressed patients with cognitive impairment.

## 1. Introduction

In the elderly, depression is regarded as a risk factor for dementia, inducing faster progression of cognitive decline and doubling the risk of subsequent AD, and AD dementia itself is often comorbid with depression [1,2,3]. To explain this entanglement, it has been hypothesized that either previous depression accelerates brain aging through stress-mediated physiological effects, or major depressive disorder and Alzheimer’s disease could share common pathophysiological mechanisms [4]. It could be difficult to tell apart these two entities solely based on clinical presentation, and this is further complicated by the high prevalence of depression resistant to usual antidepressant drugs in elderly patients [5]. Therefore, misdiagnosis is not uncommon, with up to 30% of patients with geriatric depression erroneously diagnosed with AD [4].

Moreover, geriatric depression may present with cognitive deficits partially overlapping with typical Alzheimer’s disease (AD) symptoms [4], resulting in the so-called “depressive pseudodementia” (DP). Pseudodementia is defined as a psychiatric condition masquerading as neurodegenerative disease, which is, however, reversible with the resolution of the psychiatric condition. DP is possibly the most typical psychiatric form of dementia in the elderly population, accounting for up to 4.5% of patients assessed for cognitive decline and 0.6% of patients aged 65 or older in the community setting [6]. Several clinical criteria have been proposed to identify DP, starting from a seminal article by Wells in the 1970s [7,8], but research on the topic has waned in recent years, as the validity of the construct has been called into question [6].

Nevertheless, it would be important to differentiate between the two conditions, given the potential reversibility of DP with appropriate therapy and completely different treatment options. There are currently no established biomarkers for DP, even though some studies have found frontal hypometabolism to be associated with depression in AD. However, this finding was not replicated in patients with subjective or mild cognitive impairment [1]. Moreover, frontal hypometabolism has also been associated with depression without AD [9]. Recently, a two-step approach using hippocampal volume and subsequent amyloid-PET SUV ratio thresholds has been proposed, with an accuracy of 87% in differentiating geriatric depression with mild cognitive deficits from early AD based on higher hippocampal volume and lower amyloid load. Albeit interesting, this model has not been validated yet [4].

It is generally accepted that FDG-PET has a role in discriminating the different forms of dementia. FDG-PET still retains an important role in AD work-up, and it is included as a marker of neurodegeneration in recent AD criteria [10]. The typical pattern of AD hypometabolism involves the hippocampus, the inferior parietal lobe, the lateral temporal lobe, and the posterior cingulate cortex, while the prefrontal cortex is affected to a lesser degree [11].

Lamentably, only a few studies have dealt with the FDG-PET role in differentiating DP from neurodegenerative causes of cognitive decline, with controversial results [12]. A small study by Kumar conducted in the 1990′s showed that the FDG-PET pattern of hypometabolism did not differ between geriatric depression and AD, with diffused decreased uptake, while a study by Smith found higher metabolism in frontal and parietal areas in depressed patients compared to healthy controls. In both studies, no psychotropic drugs were given at the time of scanning, but in the first study patients had a higher degree of comorbidities and cognitive impairment, which makes its conclusions questionable [5]. Another recent study found that patients with major depressive disorder without cognitive impairment exhibited decreased frontal brain metabolism compared to healthy controls [13]. Another study by the same group found increased hippocampal glucose uptake and decreased uptake in frontal, temporal, and subcortical areas [14].

That said, a panel of European experts convened that a normal FDG-PET virtually excludes a neurodegenerative cause in patients with dementia due to its high negative predictive value, while typical neurodegenerative hypometabolic patterns are a great argument against DP [12]. Therefore, recent guidelines by the European Association of Nuclear Medicine and the European Academy of Neurology recommend the use of FDG-PET to discriminate DP, in which scans should be normal or show at most a subtle frontal hypometabolism. However, the panel encouraged specific research on the topic [15]. Based on this recommendation, FDG-PET is sometimes used to prove the neurodegenerative origin of cognitive impairment in elderly patients with treatment-resistant depression.

Here, we report a case of DP in which the severe pattern of diffuse hypometabolism on FDG-PET had initially oriented his diagnosis toward AD. However, the reversibility of the metabolic abnormalities, together with the clinical recovery after successful pharmacological treatment, helped in establishing the correct diagnosis.

## 2. Case Presentation

A 74 years-old man with a history of depression, type 2 diabetes, and hyperlipemia, was evaluated for cognitive impairment at our memory clinic. He had a previous major depressive episode in his 30s; a second episode followed when he was 73, with depressed mood, extreme anxiety with obsessive ruminations, apathy, focus on self-depreciation, and somatic concerns. This last episode was initially treated with vortioxetine, but the worsening of the psychiatric symptoms and appearance of anticonservative ideation led to two E.R. admissions in the following two months. On the last of these occasions, vortioxetine was suspended, and he was started on citalopram, quetiapine, and lorazepam, without apparent benefits. Therefore, he was admitted to the Psychiatric Day Hospital of our institution, where his therapy was again modified with delorazepam 10 drops, citalopram 20 + 10 mg, quetiapine 100 mg, and lorazepam 2.5 mg, with moderate benefit on anxiety and obsessive ruminations. However, the symptoms worsened over the course of the following two months, with suspected suicidal ideation, and he was admitted to the psychiatric ward of our institution 5 months after the beginning of the current episode. During the hospitalization, memory impairment with frequent anomia was noticed, raising the suspicion of comorbid Alzheimer’s disease. Thyroid function and folates were in the normal range, while vitamin B12 was slightly reduced (119 pg/mL, reference values 180–914), but not corrected; syphilis serology was negative. At discharge, his therapy was citalopram 20 mg, quetiapine 25 + 25 + 25 mg, delorazepam 25 + 10 drops. The patient’s mood was slightly improved, without concomitant amelioration of his amnestic cognitive impairment, for which he showed poor insight. Therefore, he was referred to our memory clinic with the clinical suspect of a neurodegenerative origin of his cognitive deficits.

Upon neurological evaluation, performed 6 months after the onset of the current episode, he appeared to be in a dismissive mood, with frequent swearing and occasional mild delusions. His MMSE was 16/30 (with mild temporal disorientation, impaired delayed repetition, and executive dysfunction), while neurological examination revealed only a mildly ataxic gait.

A few weeks later he was evaluated again by a psychiatrist, who started polytherapy with duloxetine, trazodone, quetiapine, and delorazepam, with significant improvement on depressive symptoms, apathy, and functional dependency. However, self-depreciation and anxiety were still present. Interestingly, his relatives reported an improvement of his memory deficits with this new therapy.

**Figure 1:**
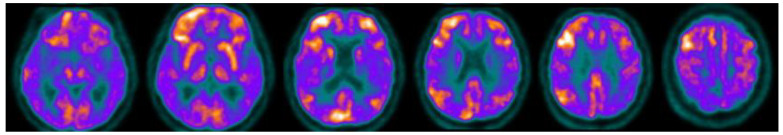
FDG-PET after the first neurological evaluation.

In the same month he underwent brain MRI, revealing only mild diffuse cortical atrophy and age-related gliosis, and brain FDG-PET, showing severe hypometabolism in bilateral precuneus, temporal, and parietal lobes (Figure 1), which was judged consistent with neurodegenerative disease, with the typical AD pattern. Notably, his glucose levels during the exam were within normal ranges (106 mg/dL), thus excluding possible artifacts due to his diabetes, which was, however, well-controlled.

Two months later, he underwent a psychometric neuropsychological evaluation (results shown in Table 1), revealing a MMSE of 30, with mild deficits in verbal and visuo-spatial long-term memory, and in interference sensitivity. His functional independence was mildly compromised for the most complex daily living activities. The psycho-affective evaluation confirmed an important mood deflection, with moderate depression, apathy, hyporexia, and mild anxiety. Possibly influenced by the knowledge of the FDG-PET results, the final neuropsychological diagnosis was initial memory impairment of likely neurodegenerative origin, in a patient with recent major depressive disorder and anxiety.

One year later, while on the same therapy for depression, he was found to be remarkably improved, and he even engaged in a few hobbies that he had previously abandoned, such as fishing and reading. He was then re-evaluated by the same neuropsychologists. Informally, mood was found to be normal. All tests were within the normal ranges, with the exception of long term visuo-spatial memory and a craving for sweets; MMSE was 28/30. The conclusion was “minimal cognitive impairment in a patient with a history of major depressive episode”.

Another year later, he was evaluated again by a psychiatrist, with tapering of the therapy for depression; the patient was now on duloxetine 60 mg, quetiapine 25 + 50 mg, trazodone 25 mg, while delorazepam was suspended. He was also taking omeprazole, metformine, aspirin, and simvastatin. At the subsequent neurological evaluation, no cognitive impairment was noticed, and his MMSE was 28/30. This was confirmed by subsequent neuropsychological tests (Table 1), on which all cognitive domains were unaffected and MMSE was 29/30. His blood routine revealed only a mild vitamin B12 deficit, with normal thyroid function, folates, and syphilis serology.

**Figure 2:**
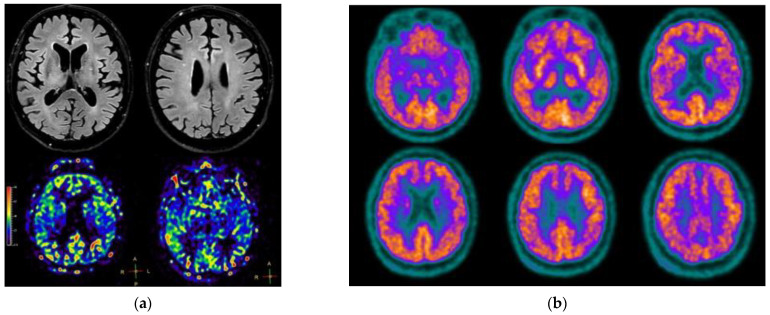
Imaging findings at last follow up: (**a**) Brain MRI—upper panels showing axial FLAIR slices, lower panel showing normal brain perfusion on axial arterial spin labeling slices; (**b**) FDG-PET.

He underwent a follow-up 3-Tesla brain MRI, showing unchanged mild cortical atrophy with normal cerebral blood flow (Figure 2a), and brain FDG-PET, surprisingly revealing a complete resolution of the previously reported hypometabolism (Figure 2b). The administered radioactivity in this case was 216 MBq, comparable with the one on the first FDG-PET, 249 MBq; the scanner used was the same, and images have been processed using the same protocol.

**Figure 3:**
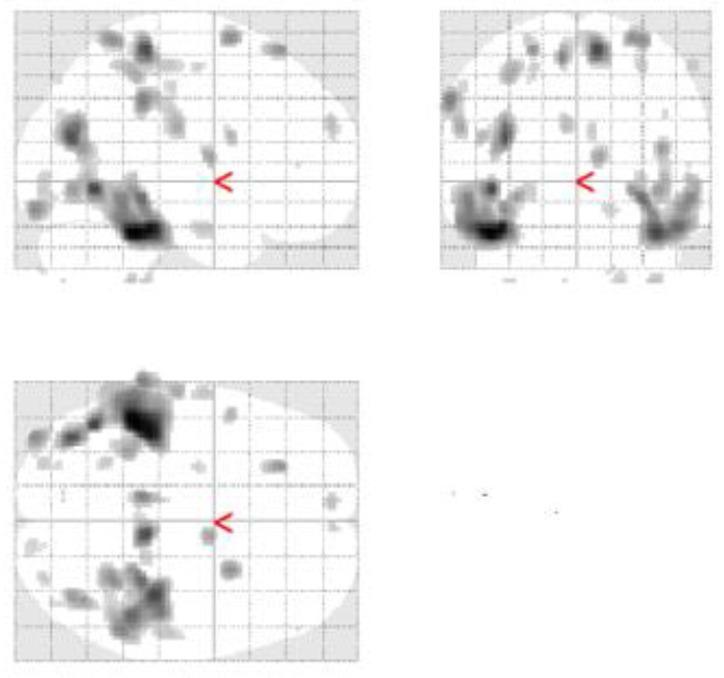
Semiquantitative analysis with SPM 12 of the first FDG-PET, showing bilateral temporo-parietal hypometabolism.

The metabolic pattern of our patient’s FDG-PET scans was evaluated firstly by an experienced rater and then verified with SPM12, implemented in the MATLAB (version R2022a), to produce maps of brain hypometabolism. A local database of 33 healthy subjects was used as controls, confirming the hypometabolism in the precuneus and parieto-temporal regions (Figure 3). Lastly, we further confirmed our findings by performing another semiquantitative analysis using the CortexID Suite (GE Healthcare, Chicage, IL, USA) using pons, whole cerebellum, and whole brain, as reference regions (data not shown).

Finally, since a lumbar puncture was judged unethical due to the absence of cognitive impairment at the time, an amyloid-PET with florbetaben was performed, showing severe cortical amyloid load in the bilateral frontal, lateral temporal, occipital lobes, precunei, cinguli, and right parietal lobe. The Brain Amyloid Plaque Score was 3/3, corresponding to a significant amyloid-β load.

## 3. Discussion

A first peculiar aspect of our case was the finding of a typical AD-like hypometabolic pattern on basal brain FDG-PET, together with a lack of the usual frontal hypometabolism, even in the context of severe treatment-resistant depression. A second surprising feature was the reversibility of such AD-like abnormalities after the improvement of depression and cognitive impairment. We discuss these two points in the next paragraphs, together with additional issues regarding the potential influence of treatment on the results of the first brain FDG-PET and the interpretation of the amyloid PET.

### 3.1. Meaning of FDG-PET Hypometabolism in Cognitive Impairment and Possible Implications

Frontal hypometabolism has been typically associated with primary and secondary depression, and with depression in the context of AD, reflecting a possibly reversible dysfunction in frontal networks underlying mood regulation [16,17].

By contrast, different FDG-PET patterns have been shown to correctly classify the major neurodegenerative dementias in more than 90% of cases [18]. In particular, in AD the typical hypometabolic pattern involving temporo-parietal lobes, precuneus, retrosplenial, and posterior cingulate cortex, has been related both to the distant effect of atrophic changes and the synaptic dysfunction caused by amyloid deposition, while metabolic compensation, mostly seen in the amygdala, has been linked to the synaptic plasticity of spared neurons [19]. It has been generally accepted that such a pattern of hypometabolism predates structural changes. However, its suboptimal specificity for AD (estimated at 84% in a recent neuropathology-confirmed series [20]) may partially explain why it was initially also observed in our case, which also suggests that considering FDG-PET as a marker of neurodegeneration in AD-like cognitive impairment might be questionable.

The reduction of cerebral metabolism may either cause or be caused by synaptic dysfunction, and it is still controversial which one precedes the other. In certain studies, a decrease in hippocampal metabolism induced subsequent AD or mild cognitive impairment (MCI), presumably mediated by a reduction of synaptic glucose consumption. This was confirmed by the observation that patients with genetic AD had reduced generalized metabolism before significant neuronal loss [21]. On the other hand, the fact that memantine, which benefits synapses, causes a reduced decline in both brain hypometabolism and cognition, might imply that synaptic dysfunction precedes glucose uptake decrease [21]. However interpreted, the entanglement of brain metabolism and synaptic function is currently well established.

Interestingly enough, in a community setting, AD-like hypometabolism and depression, or other neuropsychiatric symptoms, were associated with a higher risk of subsequent MCI development. FDG-PET abnormalities seemed to be a stronger predictor of cognitive decline, since patients with neuropsychiatric symptoms and normal FDG-PET did not have a higher risk of incident MCI, consistent with the hypothesis that synaptic dysfunction predates cognitive impairment [22].

Geriatric depression has been linked with dementia through abnormal glucose metabolism. Depression increases the risk of subsequent type 2 diabetes (notably, our patient was indeed diabetic), and fasting serum glucose (even in the normal range) in depressed patients seems to be negatively associated with brain hypometabolism in areas commonly affected by AD, while insulin resistance is positively associated with the same pattern, as well as with increased AD pathology [3].

It could be argued that brain hypometabolism contributed to the AD-like cognitive impairment seen in our case of DP, as previously reported in other similar cases (see below), and the restoration of cerebral metabolism may have influenced the subsequent cognitive improvement. These effects may have been mediated by (reversible) synaptic dysfunction rather than (irreversible) neuron loss, as previously hypothesized in AD [21]. Indeed, synaptic dysfunction has been found in depression as well, in which a lower synaptic density and functional connectivity were observed in vivo, inversely correlating with depressive symptoms and cognitive function. In the same study, synaptic loss was more coordinated in the anterior cingulate cortex and the hippocampus in patients who were more severely depressed, suggesting a possible network underlying depression and cognition [23].

If this is true, our case would suggest that restoring synaptic function would benefit brain metabolism and cognition altogether, with interesting potential consequences, which are at the moment highly speculative. For instance, considering the positive effect that electroconvulsive therapy (ECT, see below) has consistently shown on cerebral metabolism, could that be a possible treatment even for AD? Understandably, there have been no studies on humans, but a recent study on murine models of AD failed to show any effect of ECT on amyloid deposition (even though in female mice ECT resulted in a significantly lower amyloid burden at 5 weeks), neuroinflammation, or working memory. Interestingly, ECT induced temporary hippocampal neurogenesis and reduced aberrant exploratory behavior [24], consistent with studies on behavioral and psychological symptoms of dementia, for which ECT seems to be at least temporarily useful [25] (however, it must be noticed that neurogenesis has not been confirmed yet in the human brain in vivo [26]). In depressed patients responding to ECT, an increase in cerebrospinal fluid amyloid-β42 could be seen [27], which is certainly intriguing, given the relationship between depression and AD.

Another currently unanswered question is the following: if depression is indeed a risk factor for AD, could the successful treatment of DP decrease the incidence of subsequent AD development, through the restoration of cerebral metabolism and synaptic function?

The identification of further cases and the results of ad hoc studies may allow to test these intriguing hypotheses, and establish whether FDG-PET could really differentiate between DP and AD.

### 3.2. May Treatment Have Influenced the First PET?

Previous studies have shown that antidepressant therapy, in particular with paroxetine, may influence regional glucose metabolism in patients with depression in mesiotemporal and prefrontal cortices, but this finding may not directly apply to our case, since the samples involved were considerably younger and did not exhibit cognitive impairment [28,29]. Other studies in patients with geriatric depression without cognitive impairment have shown that citalopram therapy resulted in decreased cerebral metabolism in anterior cingulate, middle temporal, and parahippocampal gyri, as well as in precuneus and amygdala, with long-lasting effects at two years including hypometabolism in posterior association cortices involved in dementia, and increased metabolism in anterior cingulate cortex and insula, irrespective of treatment status at follow-up [5]. However, it is worth noticing that the longitudinal changes seen in our patients were almost the opposite, with increased uptake in the posterior region at follow-up. Moreover, our patient was on the same medications at both scans, with the only exception of delorazepam, which was discontinued. It is known that benzodiazepines may cause at most a cortical global reduction in brain metabolism due to a widespread depression of neuronal activity, but both visual assessment and SPM analysis rely on an evaluation of regional differences. Although the effect of chronic use of delorazepam on brain metabolism in humans has not yet been studied, a recent work in rats confirmed that chronic administration of diazepam, another benzodiazepine, does not alter the patterns of FDG-PET uptake [30]. Therefore, it is unlikely that treatment could have influenced the scan results in our case.

In an interesting study by Guenther, reduced vigilance evaluated by EEG influenced FDG-PET metabolism in patients with depression or MCI, with reduced uptake in frontal, temporal, and cingulate regions, and in the right thalamus [31]. However, in our case, the metabolic pattern was quite different, which would tend to rule out vigilance as a possible confounder between the two scans.

### 3.3. A Review of Reversible Brain Hypometabolism in Pseudodementia

Only a few examples of cognitive impairment with reversible brain hypometabolism after clinical improvement can be found in the literature. Most cases regard examples of psychiatric disorders presenting with cognitive decline, treated with ECT. An interesting case was reported by Bak, who described a 55 years-old woman with treatment-resistant depression and cognitive impairment. Similar to our case, she received polytherapy with duloxetine, mirtazapine, trazodone, aripiprazole, and quetiapine, but she did not significantly improve. Her FDG-PET showed diffuse brain hypometabolism (especially in frontal, parietal, and occipital areas), which completely normalized after 3 weeks of ECT, together with clinical improvement both in mood and cognition [9]. Remarkably, the reversibility of frontal hypometabolism has been already reported in patients with late-life depression treated with ECT. However, no cognitive impairment was associated in these cases [32]. Hassamal reported a 74 years-old woman with a psychotic episode and progressive cognitive impairment, showing extensive hypometabolism on FDG-PET consistent with a neurodegenerative disease, which improved after an ECT course, together with the symptoms. The authors hypothesized that the improved cerebral metabolism could represent the effect of ECT on neuropsychological symptoms, through sustained functional neural changes [18]. An analogous case was reported by Lajoie, who described a 66 years-old woman with major depression and psychotic and cognitive features, whose FDG-PET showed an AD-like hypometabolism, which reversed after ECT, together with an improvement in both psychiatric symptoms and cognition [33].

Similarly, Van Poeck reported a 74 years-old woman with a history of depression, who developed a psychotic episode with multidomain cognitive impairment during hospital admission for unrelated reasons. Her first FDG-PET showed a bilateral parietal, posterior temporal, and frontal hypometabolism, suggestive of AD. After the introduction of an unmentioned antipsychotic drug, she achieved partial recovery of her psychotic symptoms and cognitive status, together with an increase in parietal hypometabolism. Notably, a PiB-PET did not show abnormal amyloid deposition [34].

Other cases of peculiar reversible hypometabolism in patients presenting with cognitive impairment have been published. One of these cases was described by Asada, who reported a middle-aged man with alcohol-related dementia with memory impairment and depression, showing glucose hypometabolism in the right diencephalon, bilateral basal forebrain, temporal poles, supplementary motor areas, and dorsal brainstem. Together with the cessation of alcohol abuse, the patient’s clinical deficits and hypometabolism improved [35]. Interestingly, Kemppainen described a case of reversible diffuse brain hypometabolism in a patient with systemic lupus erythematosus and cognitive impairment, which improved after immunosuppressive treatment [36]. Finally, a reversible widespread brain hypometabolism in a 31 years-old patient with chronic fatigue syndrome and memory complaints treated with hyperbaric oxygen therapy for 6 weeks, was described by Mairal [37].

Based on this long list of reports, we could conclude that reversible, widespread FDG-PET hypometabolism might represent a non-specific biomarker of several functional disorders presenting with cognitive symptoms and psychiatric features, which usually require second-level therapies, falling under the broader definition of pseudodementia. That reversible metabolic alterations in the brain may produce symptoms typical of other nosological entities, which usually result from the involvement of the same regions, is further shown by a case described by Yokoyama, who reported a 67 years-old diabetic man with recurrent depressive episodes presenting with Alice-in-Wonderland syndrome, whose FDG-PET showed frontal hypometabolism and occipital hypermetabolism. After successful treatment with antidepressant therapy and ECT, both abnormalities improved [38].

On a side note, reversible brain hypometabolism can also be seen in endocrine disorders without cognitive impairment. It has been shown that hyperthyroidism resulted in brain hypometabolism, which correlated negatively with depression and anxiety severity as well, and the normalization of thyroid function partially restored cerebral glucose uptake [39].

### 3.4. Interpretation of Amyloid PET in Our Case

There is still controversy regarding AD pathology in patients with a history of major depression, with some studies reporting more pronounced AD hallmarks at autopsy and other studies not confirming these findings [1]. On the other hand, the interpretation of amyloid PET findings in the elderly population is complicated by the significant risk of false positives, since amyloid deposition is not synonymous with AD dementia. Up to 20–30% of cognitively normal patients have positive amyloid-PET (and up to 50% in ApoE4 carriers), with a delay of 15–20 years between abnormal scans and symptom occurrence [40]. Indeed, the correlation with autopsy studies for florbetaben, the tracer used in our case, showed a sensitivity of 98%, but a suboptimal specificity of 89% for AD. For our patient, even in the case of a pathological true positive, amyloid PET results could be regarded as clinical false positive, since he clearly had another established cause for his cognitive impairment, and the rigorous serial neuropsychological evaluation made it clear that the patient had completely recovered from his cognitive impairment. Incidentally, this would strengthen the validity of the clinical construct of DP, which has been called into question by some studies showing persistent cognitive deficits even after the successful treatment of depression [6]. Based on the Bayesian approach proposed by Bergeron, since the pre-test probability of AD was judged to be low in our case (as the patient did not even fulfill the criteria for MCI at the time of the scan), amyloid-PET positivity would not increase much post-test probability of AD. To provide an example, estimating a pre-test probability of 10%, the post-test probability would be only 24% (or 37% in case of ApoE4 positivity, which was not tested)—but it’s likely that these figures need to be lowered even more in our context [40].

Although the significance of amyloid PET findings was questionable (given the old age and cognitive status of our patient), since in some cases a psychiatric condition is masking an actual neurodegenerative dementia, especially when AD biomarkers are present (the so-called “pseudo-pseudodementia” [6]), the probability of our patient to eventually develop AD in the future is not null. Therefore, together with the fact that depressive symptoms are known to accelerate cognitive decline in patients with cognitive impairment and amyloid PET positivity [2], it was decided to continue to follow up the patient at least annually.

### 3.5. Learning Points and Future Directions

What we could learn from this case is that baseline FDG-PET (and probably, even amyloid PET) might not reliably differentiate between DP and AD. The pattern of hypometabolism found in typical AD largely reflects a dysfunction in the default mode network of the brain, which might be also affected by other neurodegenerative pathologies [41] or even by non-neurodegenerative conditions, as our case seem to show. Therefore, when DP is suspected, for instance, due to the rapid onset of cognitive decline together with a depressive episode, second-level psychiatric treatment could be tried to avoid placing the burden of an irreversible diagnosis on patients and their caregivers. On the other hand, repeating a PET scan after such treatment may provide evidence of the non-neurodegenerative origin of the cognitive deficits in patients with geriatric depression and AD-like cerebral hypometabolism at baseline, which would justify the validity of the DP construct and maybe provide further insight into its pathophysiology.

Rigorous studies are needed to clarify the relationship between depression and AD, unravel the role of FDG-PET in DP, and find other biomarkers for this potentially reversible cause of cognitive impairment.

In conclusion, when facing a patient with treatment-resistant depression and cognitive impairment, we should ask ourselves: is the patient resistant because he has dementia, or is the patient demented because he is resistant? Since even an established biomarker might not allow an easy answer to such a question, it could be worth trying a second-level treatment, and repeat FDG-PET scans, to identify the potential reversibility of metabolic abnormalities.

## 4. Conclusions

Our case is the first instance of DP showing an AD-like brain hypometabolism, which reverted with second-level psychiatric polytherapy, together with cognitive and mood improvement. Based on a review of reports with some similarities to ours, we argue that reversible, widespread FDG-PET hypometabolism, might represent a non-specific biomarker of severe cases of pseudodementia, which usually require second-level therapies. The pattern of metabolic dysfunction seen in our and other cases may have contributed to the AD-like cognitive presentation, and the restoration of cerebral glucose uptake with successful treatment, together with the resolution of the mood disorder and cognitive deficits, opens up intriguing scenarios. Our findings suggest caution when using FDG-PET or even pathology-specific biomarkers to differentiate between DP and AD in elderly patients with cognitive impairment and concomitant depression, as these AD biomarkers might show reduced specificity in such atypical cases, compared to the more typical amnesic presentation.

## Figures and Tables

**Table 1:** Corrected scores on neuropsychological test; pathological values are highlighted in bold.

Neuropsychological Evaluation	July 2019	July 2020	January 2022	Cut-Off Values
*Global cognitive screening*				
MMSE ^1^	30	27.86	28.86	≥23.8
*Attention and Executive Function*				
Attentional Matrices	46.25	43.25	48.25	≥41
Digit Span—backwards	4.21	4.21	5.21	≥2.65
Stroop test:				
-time interference	**54**	18.5	27	≤36.92
-errors interference	0.75	0.75	0.75	≤4.24
FAB ^1^	18	17.15	18	≥13.5
Raven Colored Progressive Matrices	24	34	35	≥18
*Short-term and long-term memory*				
Digit Span—forward	8.27	8.27	5.27	≥4.26
Rey Auditory Verbal Learning Test:				
immediate recall	39.4	50.4	44.4	≥27.98
delayed recall	**3.3**	11.3	9.3	≥4.76
Recognition	31.2	27.2	27.2	≥22.59
Recall of ROCF^1^	**8.5**	**8.5**	13.5	≥9.5
*Constructional and ideomotor apraxia*				
Copy of ROCF ^1^	29.5	35.5	34.5	≥28.9
Ideomotor apraxia (De Renzi’s test):				
right arm	70	/	68	≥53
left arm	67	/	71	≥53
*Language*				
Comprehension (subtest of ENPA ^1^):				
-Single word	20	20	19.6	≥18.4
-Sentences	14	14	14	≥11.4
Letter Fluency	35.5	29.5	44.5	≥17.78
Category Fluency	49.31	40.31	39.31	≥28.34
Picture Naming	77.89	77.89	76.89	≥67.59
*Mood and behavioral evaluation*				
Geriatric Depression Scale	**12**	**7**	**7**	<6
Neuropsychiatric Inventory:				
-depression	**8**	0	**1**	=0
-apathy	**6**	0	**1**	=0
-eating change	**6**	**6**	0	=0
-anxiety	**2**	0	0	=0
*Functional evaluation*				
ADL ^1^—completely lost functions	0	0	0	=0
ADL ^1^—partially lost functions	**1**	**1**	**1**	=0
FAQ ^1^—completely lost functions	0	**1**	**1**	=0
FAQ ^1^—partially lost functions	**3**	0	0	=0

^1^ Abbreviations: MMSE—Mini Mental State Examination; FAB—Frontal Assessment Battery; ROCF—Rey-Osterrieth Complex Figure; ENPA—Esame Neurologico per l’Afasia; ADL—Activities of Daily Living; FAQ—Functional Activities Questionnaire.

## Data Availability

All relevant data generated or analyzed during this study are included in this article. Further enquiries can be directed to the corresponding author.
